# Swarming bacteria migrate by Lévy Walk

**DOI:** 10.1038/ncomms9396

**Published:** 2015-09-25

**Authors:** Gil Ariel, Amit Rabani, Sivan Benisty, Jonathan D. Partridge, Rasika M. Harshey, Avraham Be'er

**Affiliations:** 1 Department of Mathematics, Bar-Ilan University, Ramat Gan 52000, Israel; 2 Zuckerberg Institute for Water Research, The Jacob Blaustein Institutes for Desert Research, Ben-Gurion University of the Negev, Sede Boqer Campus 84990, Midreshet Ben-Gurion, Israel; 3 Department of Molecular Biosciences, University of Texas at Austin, Austin, Texas 78712, USA

## Abstract

Individual swimming bacteria are known to bias their random trajectories in search of food and to optimize survival. The motion of bacteria within a swarm, wherein they migrate as a collective group over a solid surface, is fundamentally different as typical bacterial swarms show large-scale swirling and streaming motions involving millions to billions of cells. Here by tracking trajectories of fluorescently labelled individuals within such dense swarms, we find that the bacteria are performing super-diffusion, consistent with Lévy walks. Lévy walks are characterized by trajectories that have straight stretches for extended lengths whose variance is infinite. The evidence of super-diffusion consistent with Lévy walks in bacteria suggests that this strategy may have evolved considerably earlier than previously thought.

## Abstract (web summary)

Lévy walks have been found in the motion of large animals such as birds and fish in search of sparsely and randomly distributed food. Here, Ariel *et al*. observe, by tracking long-duration trajectories of fluorescently labelled bacteria, similar walks in bacterial swarms for the first time.

## Graphical abstract

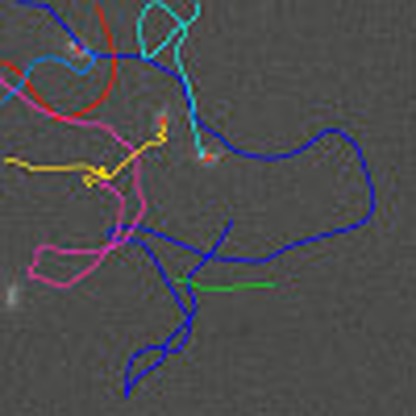

Bacterial swarming is a collective mode of motion in which flagellated bacteria migrate rapidly over surfaces^1^,^2^,^3^,^4^,^5^,^6^. Swarming is typically characterized by densely packed groups of bacteria moving in coherent swirling patterns of whirls and flows that can persist for several seconds^7^,^8^,^9^,^10^,^11^,^12^,^13^,^14^. There has been considerable recent progress in understanding the swarming phenomenon, including the underlying biological manifestations (cell elongation, increased flagellar density, secretion of wetting agents and increased antibiotic resistance)^15^,^16^,^17^,^18^,^19^,^20^,^21^, the physical interactions between cells and the medium (steric and hydrodynamic interactions, and reduction of viscosity in crowded suspensions)^7^,^8^,^9^,^10^,^22^,^23^,^24^, and the statistical properties of the swarm (distribution of group velocities, correlations and clustering)^7^,^8^,^9^,^10^,^25^,^26^,^27^. The swarm traps a water reservoir, within which individual cell speeds are comparable to swimming speeds in bulk liquid^2^,^13^,^28^,^29^. Unlike swimming bacteria, which migrate towards a nutrient source using a biased random walk controlled by a chemosensory signal transduction^30^, the continuously circling motion of individual bacteria within an expanding swarm is apparently random, undirected and independent of the chemotactic signalling system^2^.

Theoretically, it has been shown that dense suspensions of self-propelled rods are subject to orientational order instabilities which may be driving the vortex-like and irregular dynamic patterns of swarming bacteria^31^,^32^,^33^,^34^,^35^,^36^. In other words, the swirling dynamics is a physical consequence of the mechanical characteristics bacteria exhibit during swarming. None the less, this dynamical pattern seems far from an optimal, energy efficient way to get from one point in the colony to another and the evolutionary advantage of continuously circling in an apparently undirected manner during swarming is not immediately obvious. Current speculations about the advantages of this motion go beyond the simple issue of transit and transportation. For example, it has been suggested that swirling of *Bacillus subtilis* increases the mixing of oxygen inside suspensions^25^,^37^. However, these populations are much thicker than typical swarming colonies in which oxygen is not a limiting factor. In addition, swirling was suggested to play a role in prevailing against antimicrobials^15^,^38^,^39^,^40^.

Previous studies analysing the dynamical swirling patterns of swarming bacteria used one of two experimental approaches. The first used inanimate spherical beads with different diameters (0.5–10 μm) that were embedded in the colonies^8^,^19^,^41^. The results showed that at length scales of the order of the bead diameter and higher, the motion of the beads is a standard diffusion. This is not surprising, as passive beads within a dense population can be considered as Brownian particles performing a normal diffusion process. The second approach applied video analysis methods (particle image velocimetry or optical flow)^8^ to obtain either short individual trajectories (up to ∼1 s; refs 10, 13, 16) or a locally averaged velocity field describing the collective dynamics of groups and clusters. These methods cannot resolve the individual motion of bacteria to provide long-time trajectories of individuals.

In this work we report a finding that may offer an insight into the swirling patterns of the bacteria. By fluorescently labelling a subset of the motile bacteria, we have tracked individual swarming cells within their natural highly dense context, and obtained long-time trajectories at high spatial and temporal resolution. Our results reveal that the trajectories of swarming cells are super-diffusive, performing a Lévy walk (LW). The LW model is a continuous-time random walk in which particles move with a fixed speed, making sharp turns at random times with a power-law distribution^42^. As a result, these processes are characterized by trajectories that have straight stretches for extended lengths whose variance is infinite^43^,^44^,^45^. Variations, for example with a distribution of speeds, have also been studied^46^. Extensive research into the properties of LWs has led to the (still debated) hypothesis of its advantage as a foraging and exploration strategy^46^,^47^,^48^,^49^,^50^,^51^,^52^,^53^,^54^,^55^,^56^,^57^,^58^. Here we show for the first time observations of swarming bacteria performing random motion consistent with LW behaviour.

## Results

### Observation and acquisition of individual cell trajectories

**Figure 1:**
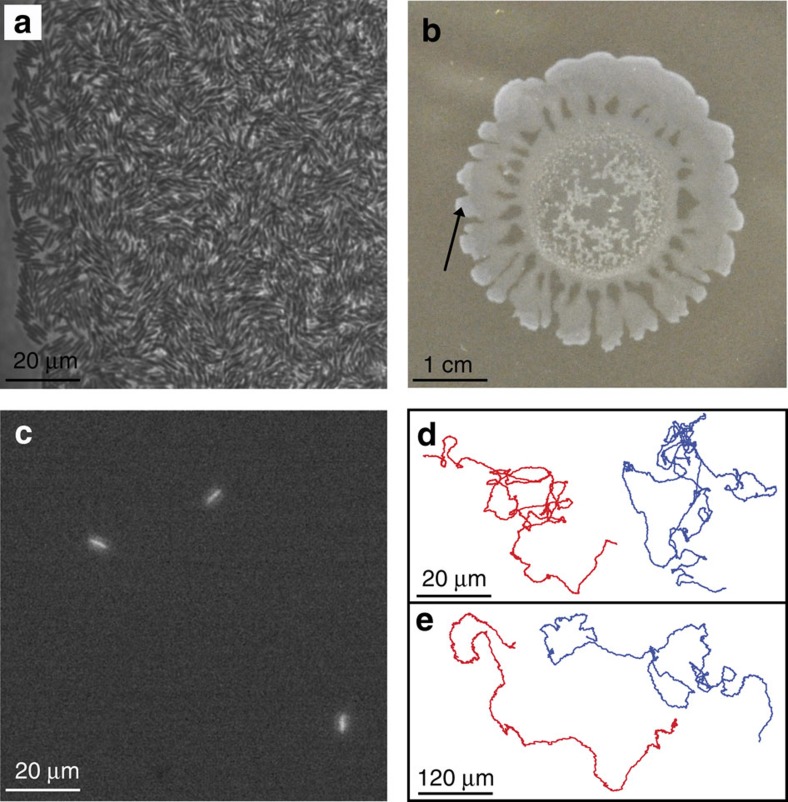
Tracking individual bacteria within a dense swarm. (**a**–**b**) Phase contrast imaging of a wild type *B. subtilis* swarming colony: at high (**a**) and low (**b**) magnifications (region of interest is marked with an arrow in (**b**)). (**c**) Fluorescent microscopy showing the fluorescently labelled bacteria only, at high magnification. (**d**–**e**) Example trajectories of individual bacteria inside the swarm at high (**d**) and low (**e**) magnifications. Left/Red: *B. subtilis* and Blue/Right: *S. marcescens*.

Fluorescently labelled *B. subtilis* cells expressing a red fluorescence protein (RFP) were mixed with unlabelled cells at a ratio of ∼1:100 and co-inoculated on swarm agar plates. The bacteria grow into a dense, motile colony, which begins expanding outward after 4 h and covers the agar plate after a further 3 h. We focused on the outer regions of the expanding swarm where the colony is three-dimensional with multiple layers (∼3 μm), and the cells are more active (Fig. 1a,b). Single cells migrating within the swarm were detected by fluorescence microscopy (Fig. 1c; Supplementary Movie 1) and their trajectories were acquired at two magnifications (Fig. 1d,e). At high (× 63) magnification, a single bacterium covers ∼1,000 pixels; this ensures a precise detection of its location and an accurate trajectory. Because the cells tend to leave the field of view within a few seconds, we repeated the experiment using a lower magnification. At low (× 20) magnification a cell covers ∼100 pixels; this magnification is less precise in resolving shorter spatial scales, however, it allows the capture of longer trajectories. Combining results from both magnifications provided accurate information on the position of the cells as a function of time as well as robust statistics. Similar experiments with a different swarming species, *Serratia marcescens*, yielded essentially the same results (blue trajectories in Fig. 1d,e).

### Trajectories are super diffusive

**Figure 2:**
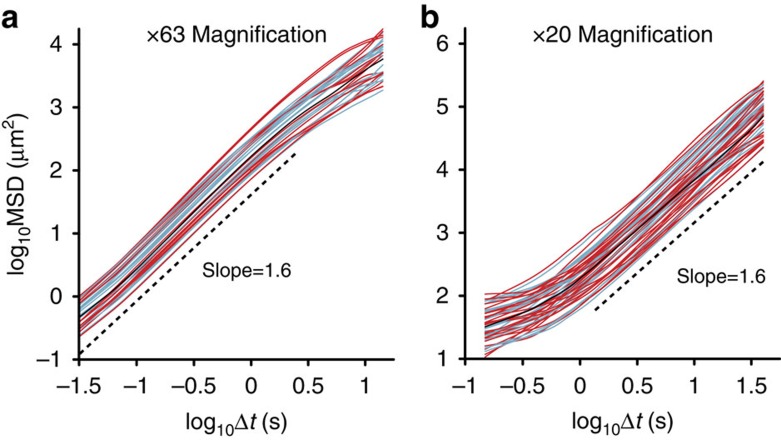
Mean square displacement of single bacteria. A slope of 1.6 is obtained for all bacteria; red lines show results with *B. subtilis* and blue lines with *S. marcescens*. The black line is the average of all bacteria. Data obtained with (**a**) high and (**b**) low magnifications.

Trajectories of individual swarming bacteria were obtained for a wide range of temporal and spatial scales: from 0.02 to 45 s and from 0.3 to 400 μm. Figure 2 shows the mean-square displacement of cells as a function of time. Above a length scale of ∼1 μm (size of cell; see Fig. 2a), all measurements show supper-diffusive dynamics with an exponent of α=1.6,


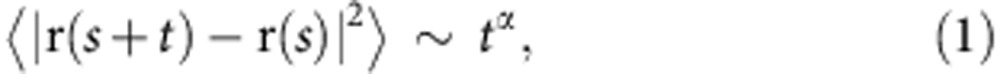


where r(*s*) denotes the location of a cell at time *s* projected on the focal plane and brackets denote averaging with respect to sample trajectories and time *s*. At longer times (>2 s), the slope reduces to ∼1. This is because cells, which were sampled during a relatively long straight stretch, have a higher probability of leaving the field of view quickly. As a result, long excursions are under-sampled. Figure 2b shows that this effect is removed at a lower magnification that allows detection of longer trajectories (∼45 s and 400 μm). Combining the two magnifications, we see that the super-diffusive behaviour persists for four orders of spatial and temporal scales. These are considerably larger time and length scales than observed in the collective jets and vortices, which persist typically for ∼0.2 s and have a scale of ∼10 μm. Beyond 400 μm, the mean-square displacements obtained by different trajectories are significantly different due to a similar bias in the sampling of long trajectories; Supplementary Fig. 1. In addition, the variance in the apparent diffusion constants (the intersection point of curves with the *y* axis) seems to be due to sampling bias rather than a property of individual bacteria; Supplementary Fig. 2.

### Trajectories are consistent with Lévy walks

**Figure 3:**
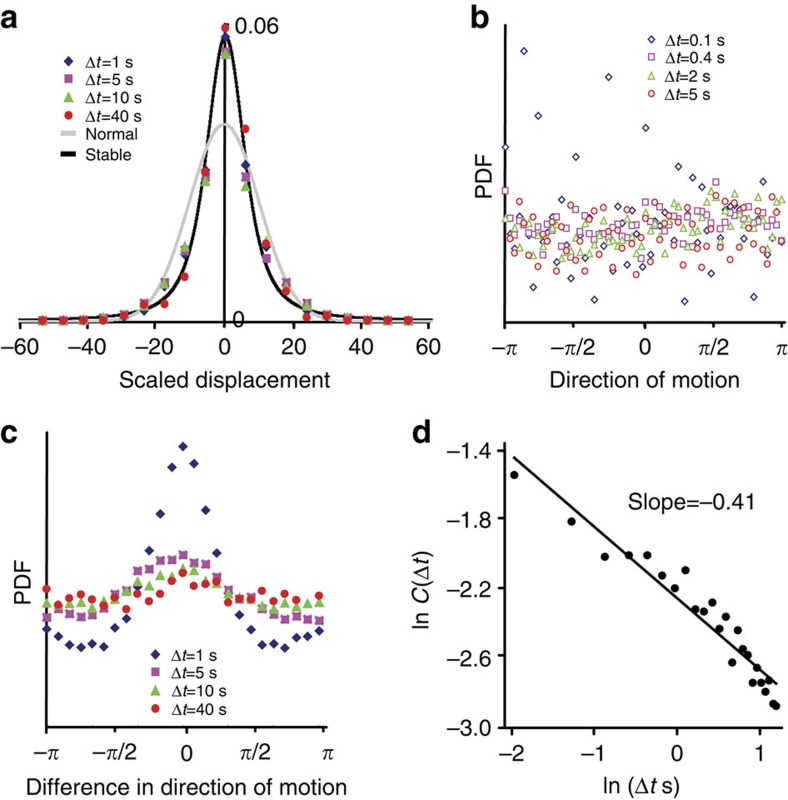
Statistics of cell displacements. Analysis of trajectories using the low magnification. (**a**) The distribution of cell displacements along each axis between a fixed number of frames. Following a scaling of Δ*t*^1/1.33^, all distributions approximately fit a Lévy stable distribution with parameter 1.33. (**b**) The probability density function (PDF) of cell directions (with respect to an arbitrary lateral axis of the frame) is uniform for all time intervals. Data were taken at high magnification. (**c**) Persistence in the direction of motion. The direction of motion remains fairly constant for times that are significantly longer than the characteristic run times in bacteria (∼1 s). Experiments with *S. marcescens* yield similar results (data not shown). (**d**) The velocity auto-correlation function decays algebraically with slope of −0.41, in agreement with the theory of LWs.

To further understand the bacterial dynamics inside the swarm and compare it with previous results of the global swarm or bead dynamics, we analysed additional aspects of the trajectories. Since the type of dynamics that cells follow is mostly governed by their long-time asymptotic properties, low magnification data, which captures longer trajectories, was used. Figure 3a shows the density of displacements (displacement of cells between a fixed number of frames), *P*(Δ*x*,Δ*t*), using the lower magnification. Assuming a scaling of displacements as Δ*t*^1/*β*^, we find that *β*=1.27 minimizes the cumulative variance between the four times depicted in Fig. 3a (Δ*t*=1, 5, 10 and 40), in agreement with the theoretical prediction of *α*+*β*=3 for LWs. In addition, the scaled displacement density fits well a symmetric Lévy stable distribution with stability parameter *β*, scale parameter 5.2 and zero location parameter (zero shift; see black line). By comparison, fitting the scaled displacement distribution to a Gaussian yields a poor approximation (grey line). However, due to insufficient sampling, it is difficult to ascertain the power-law decay of the tail (Supplementary Fig. 3). The symmetric, centralized distribution implies that there is no mean drift, which is consistent with Fig. 3b, showing a uniform distribution of directions. This verifies that there is no globally preferred direction (Supplementary Fig. 4). Figure 3c shows that the direction of motion remains fairly constant for times that are significantly longer than the characteristic run times in bacteria (∼1 s). In addition, Fig. 3d depicts the velocity auto-correlation function, 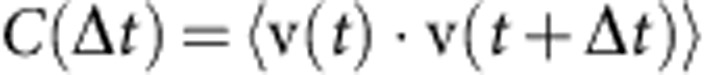 obtained with the lower magnification data. Velocities decay as Δ*t*^−*δ*^ with *δ*=0.41, in agreement with the theory of LWs, predicting that *δ*=2−α (Supplementary Note 1). The fit to an exponential is poor (Supplementary Fig. 5).

**Figure 4:**
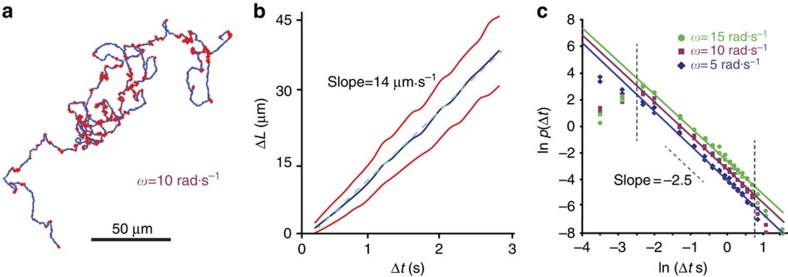
Bacterial trajectories have Lévy walk characteristics. Analysis using the high magnification. (**a**) A typical trajectory of a *B. subtilis* bacterium with turning points marked in red. Trajectory was obtained at the high magnification. (**b**) The length Δ*L* of walking segments as a function of its duration Δ*t*, indicating an approximately constant speed. A similar plot was obtained for *S. marcescens* cells with the same average speed. (**c**) Distribution of waiting times between turns showing a power-law decay with a slope of −2.5, in agreement with the theory of LWs. Filled circles, squares and diamonds show results with *B. subtilis*; empty circles, squares and diamonds show *S. marcescens*. The slope is independent of the cutoff *ω*. Straight lines are least squares fits. At short and long times (indicated by a vertical dashed line), the fit to a power-law deteriorates.

Next, we tested the hypothesis of a LW model by defining ‘turning points' in the trajectories of cells as an instant with angular speed *ω* larger than some threshold (following some smoothing of trajectories to eliminate jitter in movies; see Methods for details). As turning is a short-time and local event, high magnification data, which is captured at high temporal and spatial resolution, was used. Figure 4a shows a typical trajectory of a cell with turning points (marked in red) defined using *ω*=10 rad·s^−1^ (∼60° in 0.1 s). The distance between two consequent turns can be considered as a ‘walking segment' in a LW. Figure 4b shows the length Δ*L* of walking segments as a function of its duration, Δ*t*, indicating an approximately constant speed. The distribution of speeds within segments is plotted in Supplementary Fig. 6.

These data (Fig. 4b) imply that bacteria perform a LW rather than a Lévy flight. In contrast to the LW model, a Lévy flight is a jump process in which particle speeds vary significantly, occasionally making fast and long displacements. Trajectories of both models are indistinguishable. Indeed, Fig. 4c shows the tail of the density of segment lengths that decays like Δ*t*^−*γ*^ with *γ*=2.5 regardless of the choice of cutoff *ω*. See Supplementary Fig. 7 for results obtained using the low magnification data. Using the Akaike Information Criterion to quantitatively compare the relative likelihood of a power-law model to an exponential tail^56^ yields a weight of practically one in favour of the power-law model. This is in excellent agreement with the theory of LWs as a continuous-time random walk with constant speed, predicting that *α*+*γ*=4 (refs 59, 60).

Overall, our findings exclude stochastic models showing super-diffusion other than LW, for example, fractional Brownian motion^46^,^61^, generalized Langevin equations^46^,^62^,^63^, correlated and persistent random walks^46^,^53^,^54^,^55^,^57^,^58^,^64^, persistent random walks with variable persistence times^65^ and Lévy flights^42^,^61^. See Supplementary Notes 2–8 for supported details and simulation results in Supplementary Figs 8−13.

## Discussion

In nature, organisms face harsh conditions in which nutrients and other essential necessary resources may be depleted. In the absence of information, moving individuals resort to various random search strategies, depending on their movement abilities, the environment and the type of resources sought.

Swimming bacteria move by a process called run-and-tumble, in which short random movements (tumbles) are interspersed by long trajectories (runs). A chemotaxis signalling network encodes a short-term memory that allows the bacteria to control the length of runs and therefore bias their motion towards nutrients or away from repellents^30^. Bartumeus and Levin^66^ hypothesized that individual swimming bacteria may be performing a LW due to a power-law (with a cutoff) distribution of run times^67^,^68^,^69^. However, several recent experimental works revealed that these bacteria essentially follow a standard random walk (normal diffusion)^70^,^71^,^72^. Thus, single cells cannot improve their search strategy beyond this limitation.

We have shown that the bacteria examined in this study can use the collective dynamics of the swarm to fundamentally change the statistical properties of their dynamics. In particular, our results suggest that bacteria perform a LW. LWs were found to optimize searching in sparsely and randomly distributed targets in the absence of memory^47^,^48^. Although we cannot conclude that the random walk in our system is used as a search strategy, it is possible that swarming bacteria use the LW towards a similar end, which would imply a different foraging mechanism than that controlled by the chemosensory system during swimming. Our study shows that swarming bacteria are somehow using their large numbers to fundamentally change the statistical properties of their collective motion into a LW dynamics. This finding is in keeping with our earlier observation that MgO particles deposited on the surface of the swarm fluid display super-diffuse trajectories^19^. The high energy cost required to maintain the swirling in the swarm must be justified if it helps override the threat of death from starvation or from environmental hazards. In addition, such a behaviour has biological applications in food foraging as well as genetic and phenotypic spreading in the cases of wound repair and cancer invasion *in vitro*^73^,^74^, and for understanding fundamental aspects of how neighbouring individuals from the same population eventually end up in completely different locations.

Any physical or biological realization of a mathematical model is never precise. Accordingly, the observed super-diffusion deviates in some aspects from those of the mathematical LW. This suggests that other physical mechanisms may be important, in particular at very short or long-time scales. However, our finding suggest that in a wide range of 3–4 temporal and spatial orders of magnitude, the dynamics of swarming bacteria is consistent with a LW.

The mechanisms underlying the super-diffusive behaviour we report during swarming are fundamentally different than those hypothesized for swimming bacteria^66^,^67^,^68^,^69^. Numerous experimental and theoretical works analysing and describing the flow patterns and physical mechanisms underlying swarming show that the velocity of a swarming cell is mostly governed by the collective dynamics of the swarm and the fluid it carries rather than the precise operation of the individual flagella^8^,^9^,^10^,^11^,^12^,^13^,^14^,^15^,^16^,^17^,^18^,^19^,^20^,^21^,^22^,^23^,^24^,^25^,^26^,^27^,^28^,^29^,^30^,^31^,^32^,^33^,^34^,^35^,^36^. Thus, a bacterium does not ‘decide' to move as a Levy walker by controlling the frequency of tumbles. Instead, we suggest that it is the collective flow of the entire swarm that facilitates the LW. In this respect, the mechanism for LW is different than in other complex organisms which have been reported to follow a LW^46^,^58^. Indeed, we observe that our results are in accordance with super-diffusion reported for laminar fluid flow in a rotating annulus^75^. Solomon *et al*.^75^ suggested that particles ‘stick' to temporarily invariant surfaces around vortices for durations that exhibit power-law decays with an exponent of *γ*=2.3. The variance in the azimuthal displacement also shows a power-law growth with *α*=1.65. The agreement with our experimental results (*α*=1.6, *γ*=2.5) suggests a possible mechanism for super-diffusion in swarming bacteria—that the swirling and vortex-like patterns created by the orientational instabilities of swarming bacteria plays a similar role as the rotating two-dimensional flow and that bacteria ‘get trapped' in local vortices between extended extrusions along jets^59^. The difference between inanimate particles and swarming bacteria is that in a bacterial swarm, the turbulent velocity field is due to instabilities caused, in part, by the constant injection of energy using flagella^31^,^32^,^33^,^34^,^35^,^36^. We hypothesize that bacteria have optimized this strategy for surface translocation, perhaps by suppressing chemotaxis, and possibly to exploit it as a search strategy.

## Methods

### Bacterial strains and growth protocol

We have performed the experiments using two robust swarming bacteria, yielding essentially the same results. The first was *B. subtilis* strain 3610 (wild type), which is a Gram positive rod-shaped (0.8 × 5 μm) species, used as a model system in many quantitative swarming experiments^1^,^2^,^3^,^4^,^7^,^8^,^9^,^10^,^15^,^19^. The cells were grown on agar plates (1 g l^−1^ peptone and 0.5% agar at 30 °C); cells formed dense colonies (thickness of 3–4 μm) and began expanding outwards around 4 h after inoculation. Note that these conditions are different from published protocols that use Luria Broth (LB) and where cells swarm in a monolayer and expand out earlier^1^. A second derivative strain of 3610 was labelled with red fluorescent protein or RFP, where the protein was expressed from a chromosomal location (*ppsB*::P*trpE*-mCherry). The wild type were mixed with the RFP variant (at a ratio of 100:1) in a small tube before inoculation, then co-inoculated on swarm agar plates. Labelling does not affect swarming behaviour (Supplementary Tables 1–2). Under fluorescent microscopy only RFP cells are seen, which enables the precise detection of single cells trajectories even in a highly crowded population. The second bacterial system was *S. marcescens* strain 274 (wild type), which is a Gram negative rod-shaped (0.8 × 4 μm) species, used as a model system in previous swarming experiments^1^,^15^,^19^,^27^. The cells were grown on agar plates (LB and 0.5% agar at 30 °C); cells formed dense colonies (thickness of 3–4 μm), began expanding outwards around 5 h after inoculation and swarmed rapidly. For tracking individuals, the *S. marcescens* were labelled with a green fluorescent protein (GFP) expressed from a plasmid (pTRC99a::GFP; strain JP1020). As described above for *B. subtilis*, wild type and GFP-labelled strains were mixed at a ratio of 100:1 before inoculation on agar plates. All bacteria were stored at −80 °C in 50% glycerol stocks (antibiotics was added to frozen stocks of the RFP and GFP mutants; phleomycin for *B. subtilis* (7 μg ml^−1^) and ampicillin (100 μg ml^−1^) for *S. marcescens*) and grown overnight in LB broth at 30 °C and shaking (200 r.p.m.) before plate inoculation (5 μl at the centre of each plate).

### Observations

Optical microscopy (Zeiss Axio Imager Z2; × 20, and × 63 lenses), equipped with a sensitive high resolution video camera (NEO, Andor), was used to capture the motion of the labelled cells under fluorescence microscopy (100 frames per sec at × 63 and 7 frames per sec at × 20 and 1,000 × 1,000 pixels for both magnifications). Trajectories were obtained and analysed using Matlab. For both bacterial species, no photobleaching was observed during acquisition times (two minutes for each experiment; 6,000 frames). In each field of view we typically had ∼5 labelled cells at × 63 and ∼50 labelled cells at × 20. The total data summarizes results from tens of experiments with hundreds of cells from each species. Because standard fluorescent light strongly affects cell motility (it usually completely stops their motion in less than 1 s), we used a slightly modified version for the filters and dichroic mirror. The GFP-labelled cells were observed by standard yellow fluorescent protein Zeiss illumination setup instead of the standard GFP one (Filter set 46 yellow fluorescent protein shift free: Excitation 500/25; Beam Splitter 515; Emission 535/30). The cell intensity was slightly weaker compared to GFP. The RFP labelled cells, designed initially for mCherry illumination setup, were observed by standard Rhodamin (RFP) Zeiss illumination setup (Filter set 20 Rhodamin shift free: Excitation 546/12; Beam Splitter 560; Emission 607/80).

### Smoothing of trajectories

In order to define turning points, trajectories were smoothed using Matlab's malowess function which locally fits a polynomial (second order) to a moving window (11 frames). Then, ‘turning points' were defined as an instant with angular speed larger than a given threshold.

## Additional information

**How to cite this article:** Ariel, G. *et al*. Swarming bacteria migrate by Lévy Walk. *Nat. Commun.* 6:8396 doi: 10.1038/ncomms9396 (2015).

## Supplementary Material

Supplementary Information Supplementary Figures 1-13, Supplementary Tables 1-2, Supplementary Notes 1-8 and Supplementary References

Supplementary Movie 1 Swarming of B. *subtilis*. A low concentration (~0.2%) of fluorescently labeled motile cells is mixed with the wild type population. Fluorescent microscopy reveals the trajectories of individuals moving inside the dense swirling swarm. Frame dimensions are 100×100 μm; 63 X objective lens; real-time.
