# Supplementary material for: Swarming bacteria migrate by Lévy Walk
Source: Nat Commun. 2015 Sep 25;6:8396. doi: 10.1038/ncomms9396 (PMC4598630; doi:10.1038/ncomms9396)
Supplement: Supplementary Information — Supplementary Figures 1-13, Supplementary Tables 1-2, Supplementary Notes 1-8 and Supplementary References [file ncomms9396-s1.pdf]

Supplementary Figures

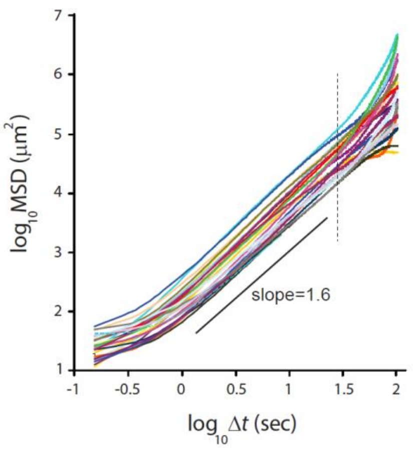

Supplementary Figure 1 | Mean Square Displacement (MSD) of single bacteria as a function of time (log-log scale) shows super-diffusive dynamics. The figure shows an extended version of Fig. 2b (low magnification), including longer temporal and spatial scales. At longer times (>30 sec indicated by a vertical dashed line), the curves diverge due to under-sampling of long excursions that leave the field of view.

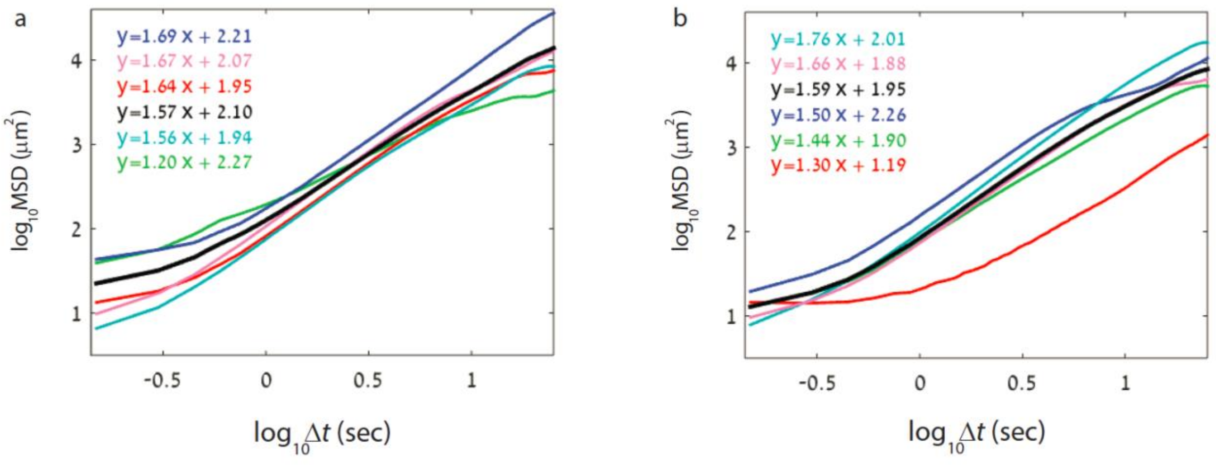

Supplementary Figure 2 | Analyzing cell variability. MSD obtained using the first and second halves of the 5 longest trajectories. Equations are the least-square fit of the tail to a line. The difference in parameters between halves demonstrates that the variability between the curves is due to sampling rather than a property of individual bacteria. Note that even with only 5 trajectories, the average fit (black curve) is accurate.

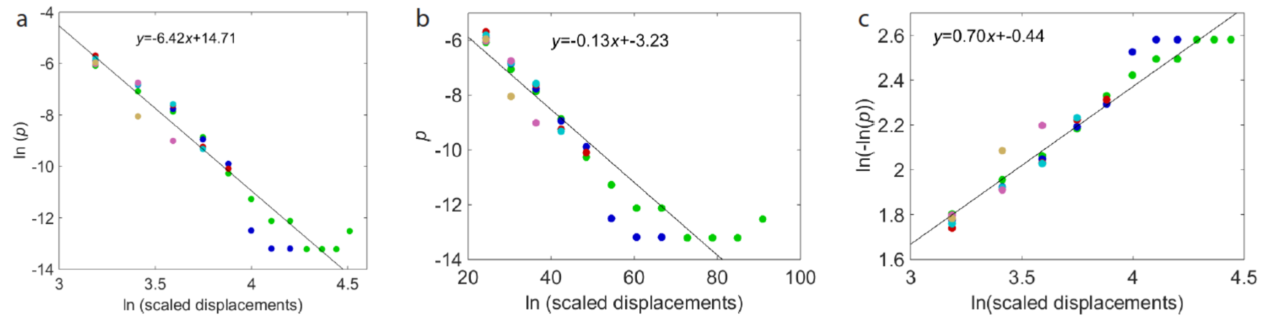

Supplementary Figure 3 | Scaled displacements on logarithmic scales. According to the theory of LWs, the scaled displacements *p* are Lévy-stable distributions. Accordingly, the tail of the distribution should decay as a power law. Here we plot the displacements on different logarithmic and semi-logarithmic scales. Due to insufficient sampling of extremely long trajectories, the fit is inconclusive.

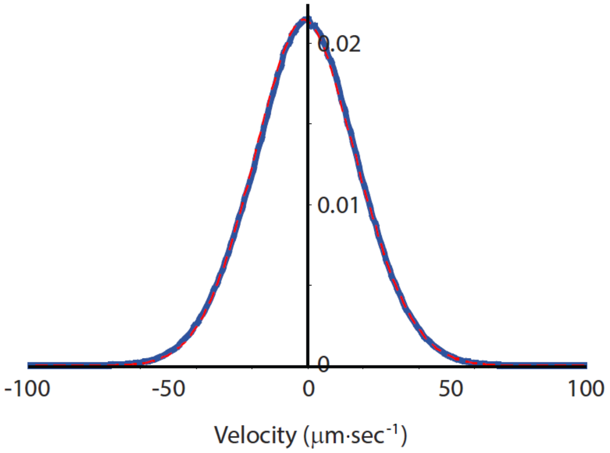

Supplementary Figure 4 | No drift or preferred flow direction in the swarm. An average drift or preferred direction should be evident in the average flow of the swarm. The figure shows the distribution of velocities of the collective swarm dynamics obtained by optical flow analysis using optical microscopy. The mean velocity in both the *x* (solid blue) and *y* (dashed red) directions is negligible (0.027 μm·sec^{-1} and 0.008 μm·sec^{-1}) with standard deviation of 19.15 μm·sec^{-1} and 19.61 μm·sec^{-1}. Data was taken for 60 sec in a single experiment with *B. subtilis*.

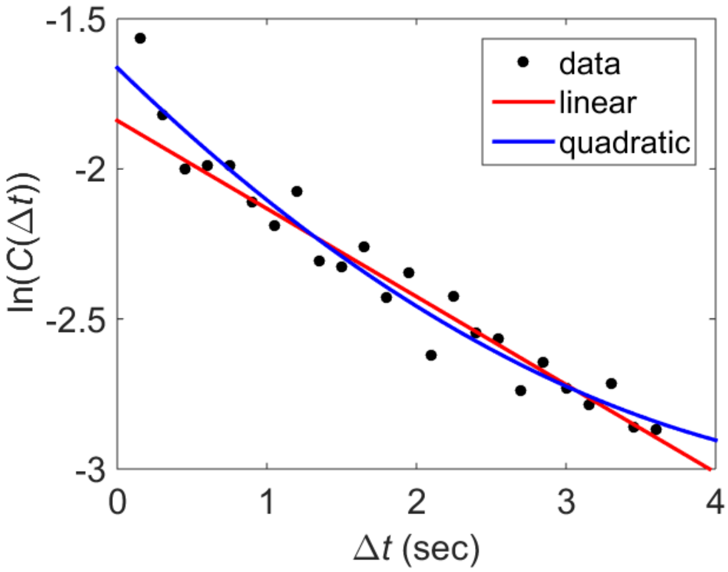

Supplementary Figure 5 | The velocity auto-correlation function, similar to Fig. 3d, plotted on a semi-log scale. A fit to a 2nd order (quadratic) polynomial shows that the curve is convex, indicating that the fit to an exponential decay is poor.

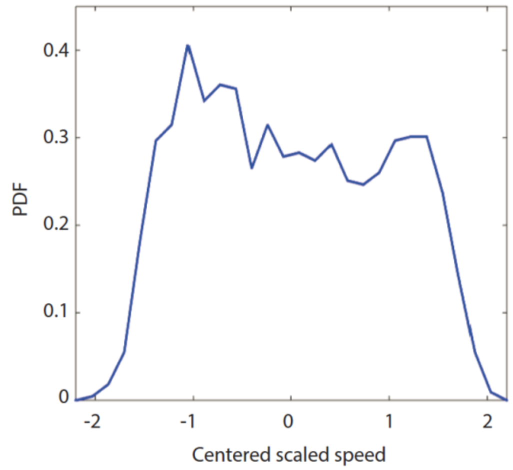

Supplementary Figure 6 | The distribution of speeds within segments. The LW model assumes that the speed in each walking segment is constant. Variable speed is not expected to qualitatively change the predictions of the model as long as the distribution of speeds has a finite variance. To this end, we calculated the centralized, scaled (zero mean and unit variance) density of speeds in each trajectory. The figure shows the average density with the 20% longest trajectories, indicating a sharp cut-off at approximately twice the standard deviation.

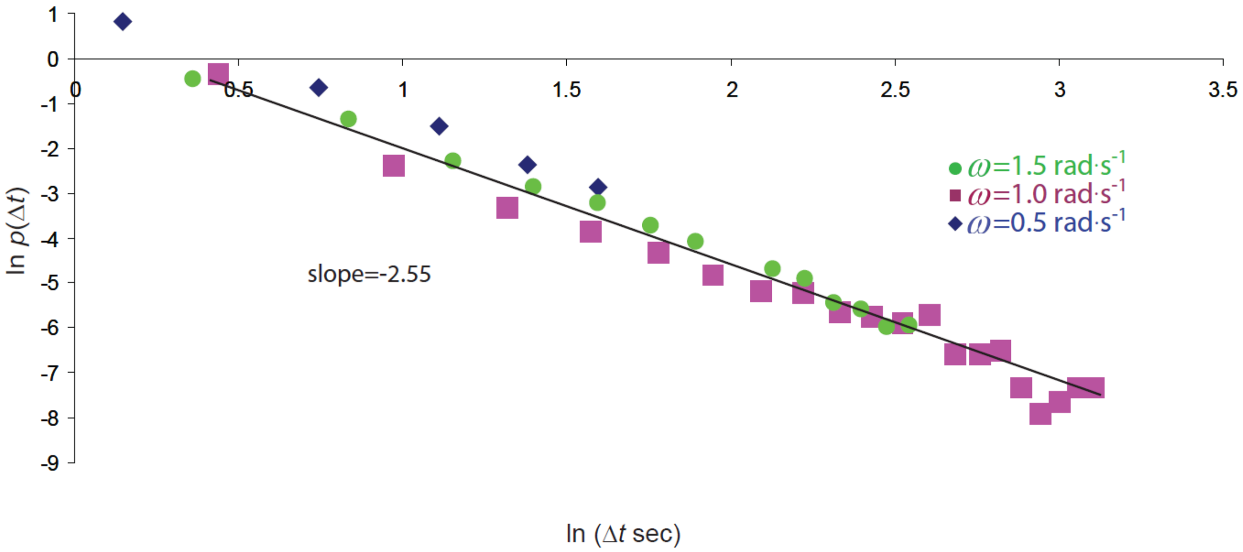

Supplementary Figure 7 | Distribution of waiting times between turns analyzed using *B. subtilis* low magnification data. The slope of -2.55 is in agreement with the theory of LWs. Straight lines are least squares fits. Due to the reduced temporal and spatial resolution of the low magnification data (see Methods), rapid turds cannot be detected.

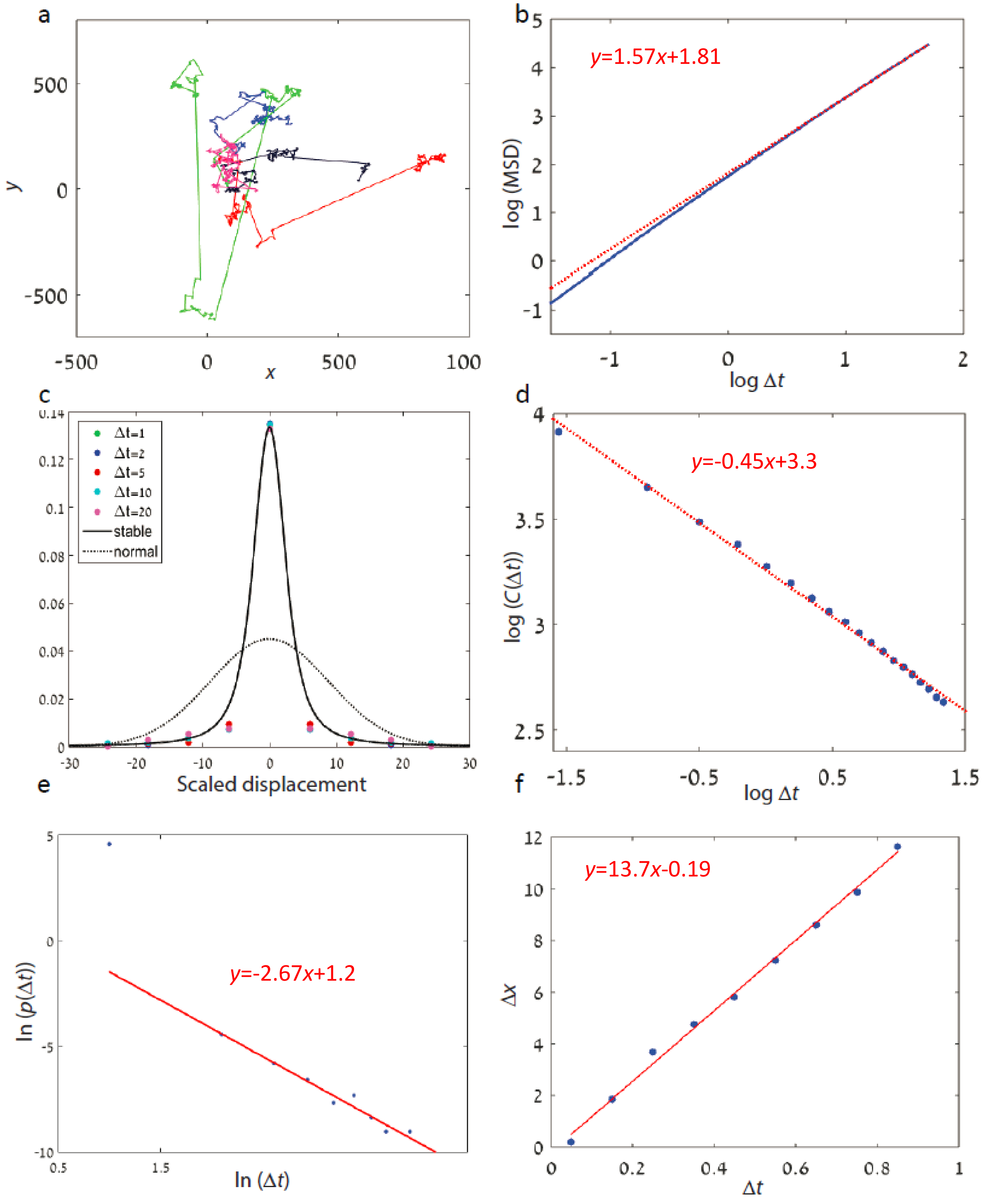

Supplementary Figure 8 | Simulation results for the LW model.

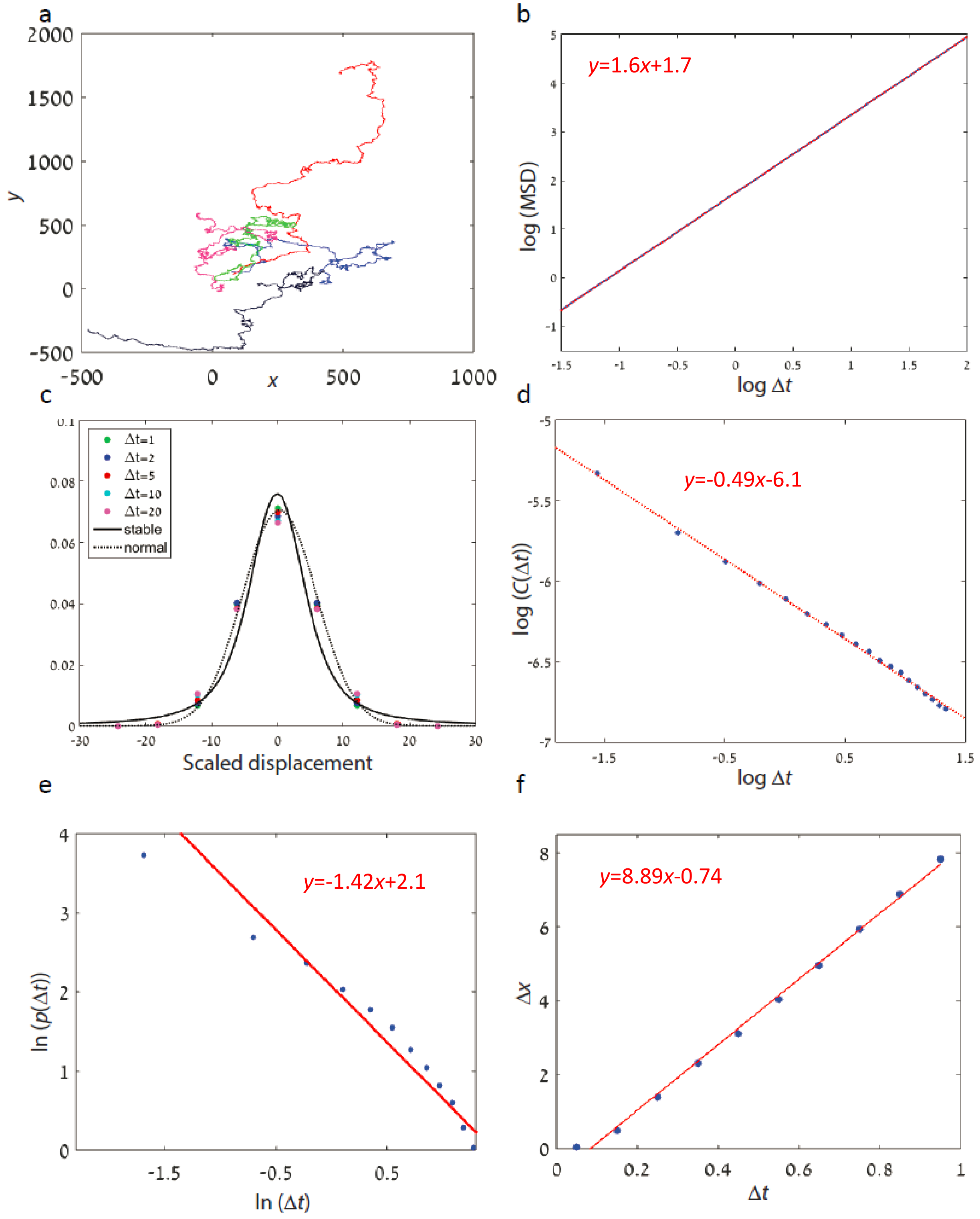

Supplementary Figure 9 | Simulation results for the fractional Brownian motion model.

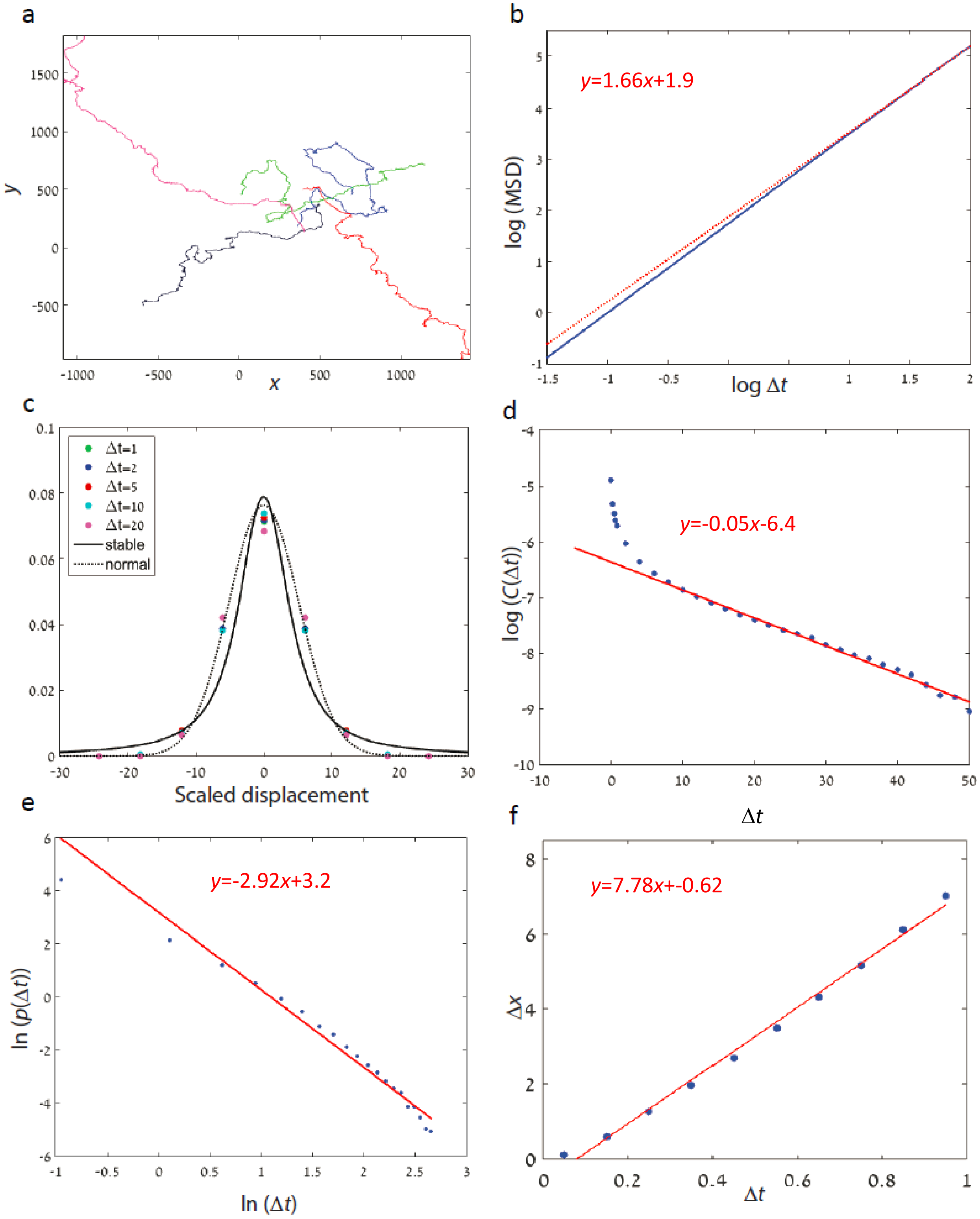

Supplementary Figure 10 | Simulation results for the generalized Langevin equation model.

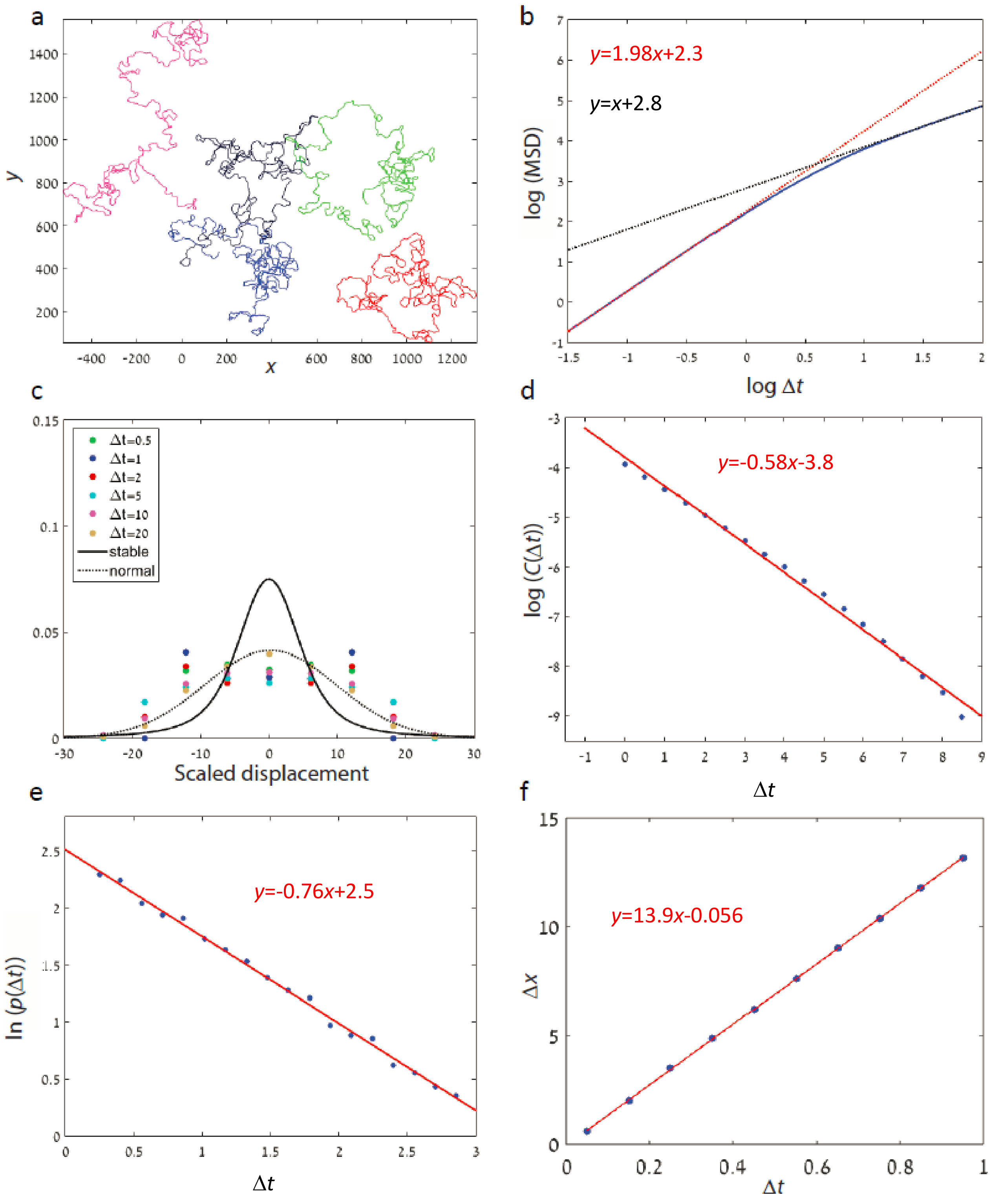

Supplementary Figure 11 | Simulation results for the correlated random walk model.

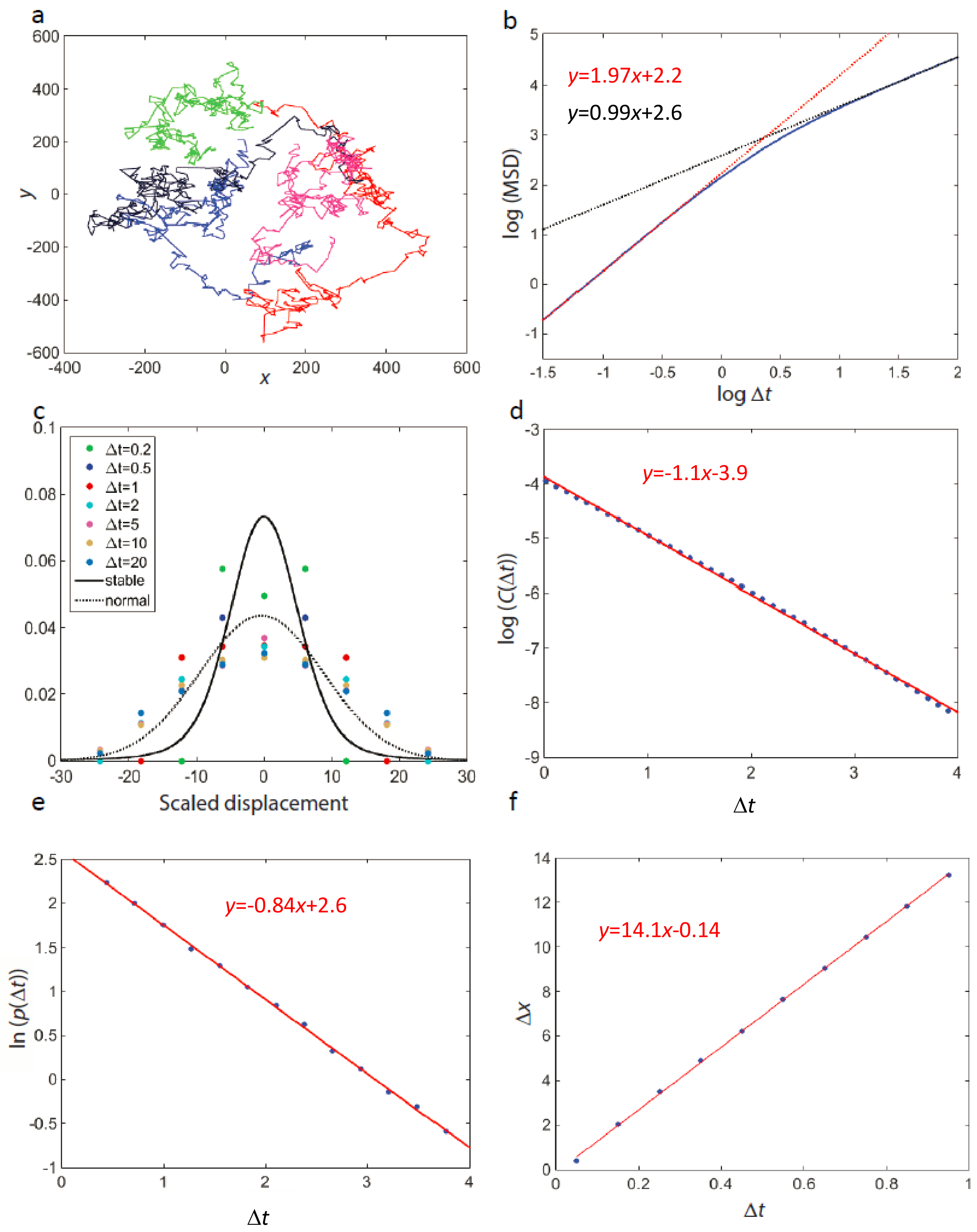

Supplementary Figure 12 | Simulation results for the persistent random walk model.

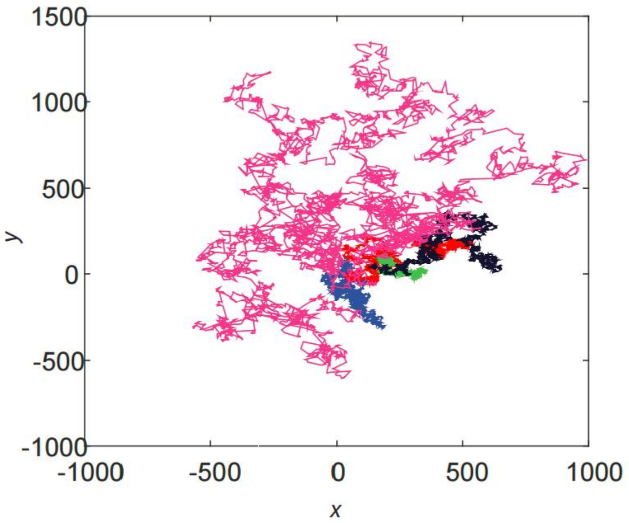

Supplementary Figure 13 | Simulation results for the persistent random walk with variable persistence times model.

Supplementary Tables

Supplementary Table 1

Comparing WT *B. subtilis* cells and RFP labeled ones, with and without fluorescent illumination. Data is presented for both collective parameters (over 10 independent experiments), and individual ones (over 100 measurements). The table shows the mean±standard deviation. The microscopic mean speed is obtained using optical flow analysis of the swarm.

		WT *B. subtilis*	RFP *B. subtilis*	WT *B. subtilis* Fluorescent light **on** for 5 min	RFP *B. subtilis* Fluorescent light **on** for 5 min
Collective	Colony expansion speed ($mm \cdot h^{-1}$)	12.4±1.2	11.5±1.7	12.3±1.3	11.7±1.6
	Microscopic mean speed ($\mu m \cdot sec^{-1}$)	24.9±2.2	25.8±2.5	25.9±1.9	24.4±2.2
	Correlation length in velocity field (μm)	3.2±0.5	3.4±0.8	3.2±0.6	3.3±0.5
	Correlation time in velocity field (sec)	0.16±0.05	0.15±0.04	0.13±0.06	0.15±0.06
Individual	Swimming run speed in broth ($\mu m \cdot sec^{-1}$)	32.1±4.2	30.5±3.5	32.3±3.1	33.8±4.0
	Time between tumbles in broth (sec)	5.2±3.6	4.9±2.8	5.0±4.2	5.1±3.0

Supplementary Table 2

Comparing WT *S. marcescens* cells and GFP labeled ones, with and without fluorescent illumination. Data is presented for both collective parameters (over 10 independent experiments), and individual ones (over 100 measurements). The table shows the mean±standard deviation. The microscopic mean speed is obtained using optical flow analysis of the swarm.

		WT *S. marcescens*	RFP *S. marcescens*	WT *S. marcescens* Fluorescent light **on** for 5 min	GFP *S. marcescens* Fluorescent light **on** for 5 min
Collective	Colony expansion speed ($mm \cdot h^{-1}$)	9.1±1.6	8.3±1.4	8.2±1.8	8.5±1.9
	Microscopic mean speed ($\mu m \cdot sec^{-1}$)	19.3±2.8	18.1±2.0	18.8±2.4	20.3±2.3
	Correlation length in velocity field (μm)	3.7±0.8	3.9±0.4	3.6±0.7	3.5±0.5
	Correlation time in velocity field (sec)	0.28±0.08	0.25±0.06	0.32±0.08	0.26±0.08
Individual	Swimming run speed in broth ($\mu m \cdot sec^{-1}$)	19±4.4	20±3.7	18±3.2	22±4.1
	Time between tumbles in broth (sec)	2.2±1.5	2.0±1.2	2.4±1.9	2.1±1.1

Supplementary Notes

Supplementary Note 1: The velocity auto-correlation function for LWs.

Let $\mathrm{v}(t)$ denote the velocity at time t of a particle following a LW. In the original LW model [1], particles move at constant speed between random reorientations. Without loss of generality, we assume unit speed. We assume that ψ has a finite average.

We define the velocity auto-correlation function as

(1) $$C(\Delta t) = \langle \mathrm{v}(t)\mathrm{v}(t+\Delta t)\rangle,$$

where $\langle\cdot\rangle$ denotes averaging over all times t in independent samples of infinite trajectories. Since following a reorientation event a particle completely losses its memory, we have that $C(t) = P(NJ(t,t+\Delta t))$, where $NJ(t,t+\Delta t)$ is the event that there is not jump in $[t,t+\Delta t]$. Next, let $l(\tau)$ denote the event that a randomly sampled time is inside a jump of length τ. Formally, $P(l(\tau)) = \tau\psi(\tau)dt / < \tau >$. Using the total probability theorem,

(2) $$C(t) = P(NJ(t,t+\Delta t)) = \int_0^\infty P(l(\tau))\langle NJ(t,t+\Delta t)\,|\,l(\tau)\rangle d\tau .$$

It is clear that

(3) $$\langle NJ(t,t+\Delta t)\,|\,l(\tau)\rangle = \begin{cases} 0 & \tau \le \Delta t \\ (\tau-\Delta t)/\tau & \tau > \Delta t. \end{cases}$$

This implies that

(4) $$C(t) = \frac{1}{\langle\tau\rangle}\int_{\Delta t}^\infty (\tau-\Delta t)\psi(\tau)d\tau .$$

For example, if $\psi(\tau)$ is an exponential distribution with rate λ, Eq. (4) yields the expected $C(t) = e^{-\lambda t}$. Assuming waiting times between reorientation events have a density $\psi(\tau)$ a power-law tail, $\psi(\tau) \sim \tau^{-\beta+1}$, yields $C(\Delta t) \sim t^{-(\beta-1)}$. See also [2].

In our experiments, $\alpha = 1.6$, $\beta = 4-\alpha = 2.4$. Therefore, $C(\Delta t)$ should have a tail with exponent -0.4.

Supplementary Note 2: Comparing models of super diffusion - Lévy walk:

We simulated six different models showing super-diffusion: LW, fractional Brownian motion [3-4], generalized Langevin equations [3, 5-6], correlated and persistent random walks [3, 7-12], persistent random walks with variable persistence times [13] and Lévy flights [1, 4]. In each simulation, 150 trajectories were sampled at a rate of 100 fps (similar to the high magnification data) and for 500 seconds (similar to the low magnification data). Parameters were chosen to match the MSD plot (Fig. 2), in particular the asymptotic slope (1.6) and the point of intersection with the *y*-axis (about -0.5 with the high magnification). Trajectories were analyzed using the same methods applied in generating Figs. 3 and 4. Thus, the experimental results can be compared with the different model predictions. All simulations were performed in Matlab.

The model: The LW model is a Continuous Time Random Walk (CTRW) in which a particle moves with a constant speed v. At randomly drawn turning events $\tau_1, \tau_2, \ldots$ the particle draws a new random orientation which is independent and uniformly distributed in $[-\pi, \pi]$. In the classical LW model [1], waiting times between turns, $\tau_{i+1} - \tau_i$, are independent and identically distributed (IID) random variables (RV) with a density $\psi(t)$ that has a power-law tail, $\psi(t) \sim t^{-\gamma}$.

Simulation method: IID waiting times were drawn from a distribution with density

(5)
$$\psi(t) = \begin{cases} t^{-\gamma} & t \geq t_{\min} \\ 0 & t < t_{\min}. \end{cases}$$

Thus, a sequence of a particle's velocity and position at the turning events can be calculated exactly. Using linear interpolation (which is, in this case, exact), a discretized trajectory at uniform time intervals with $\Delta t = 0.01$ sec? was generated. See Supplementary Figure 7a for example of sample trajectories.

Simulation parameters: $\gamma = 1.3$ and $v = 14$.

Results: See Supplementary Figure 7. The theoretical asymptotic slope of the MSD curve on a log-log plot is 1.7. However, due to sampling errors, it is slightly smaller (which is why we took $\gamma = 1.3$ and not 1.4). The optimal scaling of displacements (see the text for the optimization method), depicted in Supplementary Figure 7c, was 1.3, in agreement with theory. The scaled curves fit well a Lévy stable distribution with same parameter γ and scale factor 2.2. Note that the scale parameter is smaller than the experimentally observed value (5.2). This may be due to noise stemming from movie jitter which may affect time scales of the order of the sampling

rate (0.01 secs). The velocity auto-correlation function (Supplementary Figure 7d), distribution of waiting times (Supplementary Figure 7e) and mean speed between turns (Supplementary Figure 7f) match well both the theoretical predictions (see text) and the experimental results.

Supplementary Note 3: Comparing models of super diffusion - Fractional Brownian motion:

The model: Fractional Brownian motion (fBM), introduced by Mandelbrot & van Ness [4] is a continuous time Gaussian process, B_t^H, with covariance function

(6) $$E[B_t^H B_s^H] = \frac{D}{2}\left(|t|^{2H} + |s|^{2H} - |t| - s^{2H}\right),$$

where $E[\cdot]$ denotes expectation with respect to sampled trajectories, $H \in (0,1)$ is called the Hurst index ($H = 1/2$ is the standard Wiener process) and $D > 0$ is a diffusion constant.

Simulation method: See ref. [14]. See v Supplementary Figure 8a for example of sample trajectories.

Simulation parameters: $H = 0.8$ and $D = 40$, chosen to fit Fig. 2.

Results: See Supplementary Figure 8. The asymptotic slope of the MSD curve on a log-log plot is, as expected by the theoretical prediction of $2H$. The optimal scaling of displacements was 1.7, which is very close to the theoretical prediction of $2H = 1.6$. Since fBM is a Gaussian process, displacements have a normal distribution, which can be readily observed in Supplementary Figure 8c. The velocity auto-correlation function (Supplementary Figure 8d) is also a power law with the correct slope of $-(\gamma - 1)$ [4]. Due to the non-differentiability of fBM trajectories, turning events are not well defined. First, the width of smoothing required to identify them was larger by a factor of 10 compared to both experiments and the LW model. In addition, the distribution of waiting times (Supplementary Figure 8e) and mean speed between turns (Supplementary Figure 8f) do not match the experimental results.

Supplementary Note 4: Comparing models of super diffusion - Generalised Langevin equation:

The model: Generalised Langevin equation (GLE) is a non-Markovian generalization of the well-known Langevin dynamics [6, 15]. The generalization is obtained by introducing a singular power-law memory kernel. Formally, in 1D the GLE is given by a 2nd order SDE,

(7) $$\ddot{x} + \int_{-\infty}^{t} \eta(t-s)\dot{x}(s)ds + \nabla V(x,t) = \xi(t),$$

where, $\eta(t)$ is a memory kernel, $V(x,t)$ is an external potential (zero in our case) and $\xi(t)$ is a stochastic driving force with zero mean and covariance

(8) $$E\left[\xi(t)\xi(s)\right] = D\eta(|t-s|).$$

The relation between the memory kernel and the covariance of noise is called a fluctuation dissipation theorem, which is a property of thermodynamic equilibrium. We note that the system of swarming bacteria is not in thermodynamic equilibrium due to the constant injection of energy by the bacterial self-propulsion mechanism.

If the spectrum of $\eta(t)$, has a power law growth at low frequencies, $\eta(\omega) \sim \omega^r$, then trajectories have a MSD that grows asymptotically as $2Dt^r / \Gamma(1+r)$. Hence, with $1 < r < 2$, the dynamics is super-diffusive.

Simulation method: See ref. [15]. See Supplementary Figure 9a for example of sample trajectories.

Simulation parameters: $r = 1.6$ and $D = 40$, chosen to fit Fig. 2.

Results: See Supplementary Figure 9. The asymptotic slope of the MSD curve on a log-log plot is, as expected by the theoretical prediction. The optimal scaling of displacements was 1.12, which is lower than the experimental results. Since the GLE noise is a Gaussian process, displacements have a normal distribution, as observed in Supplementary Figure 9c. The velocity auto-correlation function (Supplementary Figure 9d) decays exponentially. Similar to fBM, turning events are not well defined. First, the width of smoothing required to identify them was large compared to both experiments and the LW model (by a factor of 3-4). In addition, the distribution of waiting times (Supplementary Figure 9e) and mean speed between turns (Supplementary Figure 9f) do not agree with the experimental results.

Supplementary Note 5: Comparing models of super diffusion - Correlated random walk:

The model: Correlated random walk (CRW) is a Markovian discrete random walk process in which increments are correlated. In 2D,

(9)
$$X_n = \sum_{i=1}^{n} \Delta X_i ,$$

where, $|\Delta X_i| = v_i \Delta t$, Δt is the simulation time step and the velocity at step i, v_i has a fixed norm v. The angles between consecutive increments, $\Delta\theta_i$, $\cos\Delta\theta_i = (v_i \cdot v_{i-1}) v^{-2}$ are IID with a given (even) distribution in $[-\pi, \pi]$ [16-17].

Simulation method: $\Delta\theta_i$ is drawn from the von-Misses distribution with zero mean and concentration parameter κ. See Supplementary Figure 10a for example of sample trajectories.

Simulation parameters: $v = 14$ and $\kappa = 1/\Delta t$, which implies a persistence time of about 1 sec, a typical run length for swimming bacteria.

Results: See Supplementary Figure 10. In agreement with theoretical predictions [17], the slope of the MSD curve on a log-log plot changes from 2 for short time scales (ballistic motion) to 1 on long scales (normal diffusion). The transition to a diffusive behavior occurs at around $\Delta t = 2$ secs. This is in contrast to the experimental observations showing anomalous diffusion up to 40 secs. The optimal scaling of displacements was 1.6, which is in agreement with the experimental results. However, the scaling of different time steps does not all fall nicely onto a single master curve. Since the CRW is a Gaussian process, displacements have a normal distribution, as observed in Supplementary Figure 10c. The velocity auto-correlation function (Supplementary Figure 10d) decays exponentially. In addition, the distribution of waiting times (Supplementary Figure 10e) is also exponential, unlike the power-law behavior in observed experiments.

Supplementary Note 6: Comparing models of super diffusion - Persistent random walk:

The model: Persistent random walk (PRW) is a CTRW in which a particle moves with a constant speed v. At randomly drawn turning events $\tau_1, \tau_2, \ldots$the particle draws a new random orientation which is independent and uniformly distributed in $[-\pi, \pi]$. However, unlike in the LW model, waiting times between turns, $\tau_{i+1} - \tau_i$, are IID exponential RVs [18].

Simulation method: At every simulation step, a particle has probability $P\Delta t$ to make a random turn, where P is the persistence length and Δt is the simulation time step. See Supplementary Figure 11a for example of sample trajectories.

Simulation parameters: $v = 14$ and $P = 1\sec^{-1}$, which implies a persistence time of 1 sec, a typical run length for swimming bacteria.

Results: See Supplementary Figure 11. Results are similar to CRW. In agreement with theoretical predictions [17], the slope of the MSD curve on a log-log plot changes from 2 for short time scales (ballistic motion) to 1 on long scales (normal diffusion). The transition to a diffusive behavior occurs at around $\Delta t = 2$ secs. This is in contrast to the experimental observations showing anomalous diffusion up to 40 secs. The optimal scaling of displacements was 1.3, which is slightly lower than the experimental results. Moreover, the scaling of different time steps does not all fall nicely onto a single master curve. Since the PRW is a Gaussian process, displacements have a normal distribution, as observed in Supplementary Figure 11c. The velocity auto-correlation function (Supplementary Figure 11d) decays exponentially. In addition, the distribution of waiting times (Supplementary Figure 11e) is also exponential, unlike the power-law behavior in observed experiments.

<u>Supplementary Note 7: Comparing models of super diffusion - Persistent random walk with variable run times:</u>

The model: Petrovskii et al [13] considered a system of many non-identical particles. In this work they assumed that all particles follow a normal diffusion. However, the diffusion constant of each particle may be different. In that work it was shown that assuming that diffusion constants are IID with a power-law tail can lead to the appearance of super-diffusive MSD curves. See Supplementary Figure 12 for example of sample trajectories.

However, our experimental results show that the apparent distribution of effective diffusion constants, as observed in the different intersections with the *y*-axis of the MSD curves of different bacteria (Fig. 2a) has a compact support. In particular, it cannot have a power-law distribution. In addition, the variation between cells seems mainly due to sampling errors than a property of individual bacteria (Supplementary Figure 2).

Supplementary Note 8: Comparing models of super diffusion - Lévy flight:

A Lévy flight is a jump process in which particle speeds vary significantly, occasionally making fast and long displacements. Trajectories of both models (Lévy walk) are indistinguishable. See Supplementary Figure 7a for example of sample trajectories. The MSD of a Lévy flight diverges. In addition, Fig. 4b suggests an approximately constant speed, which in contradiction to the assumptions of a Lévy flight.

Supplementary References

[1] Shlesinger, M. F., Klafter, J. & Wong, Y. M. Random Walks with Infinite Spatial and Temporal Moments. *J. Stat. Phys*. **77**, 499-512 (1982).

[2] Bouchaud, J.P.l., Georges, A., Anomalous diffusion in disordered media. Physics Reports 195: 127-293 (1990).

[3] Metzler, R., Jeon, J. H., Cherstvy, A. G. & Barkai, E. Anomalous diffusion models and their properties: non-stationarity, non-ergodicity, and ageing at the centenary of single particle tracking. *Phys. Chem. Chem. Phys.* **16,** 24128-24164 (2014).

[4] Mandelbrot, B. & van Ness, J. W. Fractional Brownian motions, fractional noises and applications, *SIAM Review* **10**, 422-437 (1968).

[5] Eule, S., Zaburdaev, V., Friedrich, R. & Geisel, T. Langevin description of superdiffusive Lévy processes. Phys. Rev. E. 86, 041134 (2012).

[6] Fox, R. F. The generalized Langevin equation with Gaussian fluctuations. *J. Math. Phys.* **18**, 2331 (1977).

[7] Reynolds, A. M. & Rhodes, C. J. The Lévy flight paradigm: random search patterns and mechanisms. *Ecology* **90**, 877-887 (2009).

[8] Reynolds, A.M. Bridging the gulf between correlated random walks and Lévy walks: autocorrelation as a source of Lévy walk movement patterns. *J. R. Soc. Interface* **7**, 1753-1758 (2010).

[9] Harris, T.H. *et al*. Generalized Lévy walks and the role of chemokines in migration of effector CD8+ T cells. *Nature* **486**, 545-549 (2012).

[10] Codling, E. A., Plank M. J., and Benhamou, S. Random walk models in biology. *J. R. Soc. Interface* **5,** 813-834 (2008).

[11] Benhamou, S. How Many Animals Really Do the Lévy Walk? *Ecology* **88** 1962-1969 (2007).

[12] Renshaw, E. & Henderson, R. The Correlated Random Walk. *J. App. Prob.* **18**, 403-414 (1981).

[13] Petrovskii, S., Mashanova, A., & Jansen, V. A. (2011). Variation in individual walking behavior creates the impression of a Lévy flight. *Proc. Natl. Acad. Sci. U.S.A.* **108**, 8704-8707.

[14] Kroese, D.P. and Botev, Z.I. (2013). "Spatial Process Generation." V. Schmidt (Ed.). Lectures on Stochastic Geometry, Spatial Statistics and Random Fields, Volume II: Analysis, Modeling and Simulation of Complex Structures, Springer-Verlag, Berlin.

[15] Siegle, P., Goychuk, I. and Hanggi, P. Markovian embedding of fractional supersiffusion, Europhysics Lett., 93(2), 20002. (2011).

[16] Dunn, G. A., & Brown, A. F. (1987). A unified approach to analysing cell motility. Journal of Cell Science, 1987(Supplement 8), 81-102.

[17] Bovet, P., & Benhamou, S. (1988). Spatial analysis of animals' movements using a correlated random walk model. Journal of theoretical biology, 131(4), 419-433.

[18] Othmer, H. G., Dunbar, S. R., & Alt, W. (1988). Models of dispersal in biological systems. Journal of mathematical biology, 26(3), 263-298.
